# Supplementary material for: Gene Expression Signatures of a Preclinical Mouse Model during Colorectal Cancer Progression under Low-Dose Metronomic Chemotherapy
Source: Cancers (Basel). 2020 Dec 26;13(1):49. doi: 10.3390/cancers13010049 (PMC7795790; doi:10.3390/cancers13010049)
Supplement: Supplementary file 1 [file cancers-13-00049-s001.zip › cancers-1037071-suppl-XML/Figures S1-S8 final.docx]

Supplementary Materials: Gene Expression Signatures of a Preclinical Mouse Model during Colorectal Cancer Progression
under Low-Dose Metronomic Chemotherapy

| **Publisher’s Note:** MDPI stays neutral with regard to jurisdictional claims in published maps and institutional affiliations.  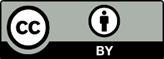  **Copyright:** © 2020 by the authors. Licensee MDPI, Basel, Switzerland. This article is an open access article distributed under the terms and conditions of the Creative Commons Attribution (CC BY) license (http://creativecommons.org/licenses/by/4.0/). |
| --- |

Hung Ho-Xuan, Gerhard Lehmann, Petar Glažar, Foivos Gypas, Norbert Eichner, Kevin Heizler, Hans J Schlitt, Mihaela Zavolan, Nikolaus Rajewsky, Gunter Meister & Christina Hackl


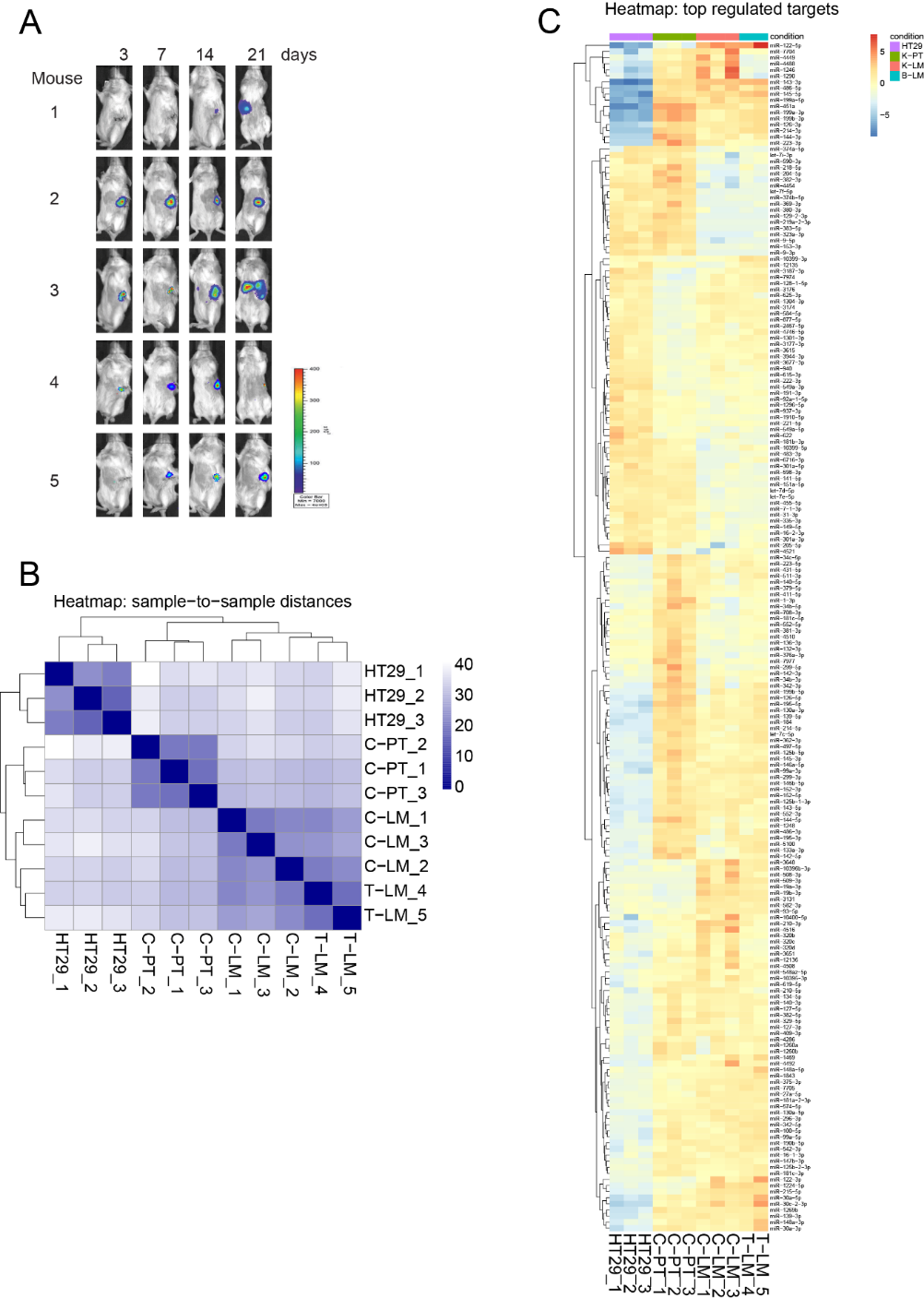


**Figure S1.** Mice used in this study and profiling of miRNAs. (**A**) Mouse preparation for development of primary tumor and liver metastasis in control and treated group. In vivo bioluminescence monitoring of intrasplenic HT29 tumors. Three days after injection, primary tumors developed. For bioluminescence imaging, mice were intraperitoneally injected with 150 mg/kg of luciferin and imaged weekly. All mice were sacrificed when bioluminescence levels of the control group reached about 500,000 photons/s. Five mice were used for this experiment. Three mice: #1, #2, #3 belong to control group while two mice: #4, #5 belong to LDM topotecan treated group. (**B**) Heatmap showing sample-to-sample distances from miRNA library. (**C**) Heatmap of the top regulated miRNAs differentially expressed in HT29 compared to primary tumor from control group (C-PT), liver metastasis from control group (C-LM) and liver metastasis from treated group T-LM.


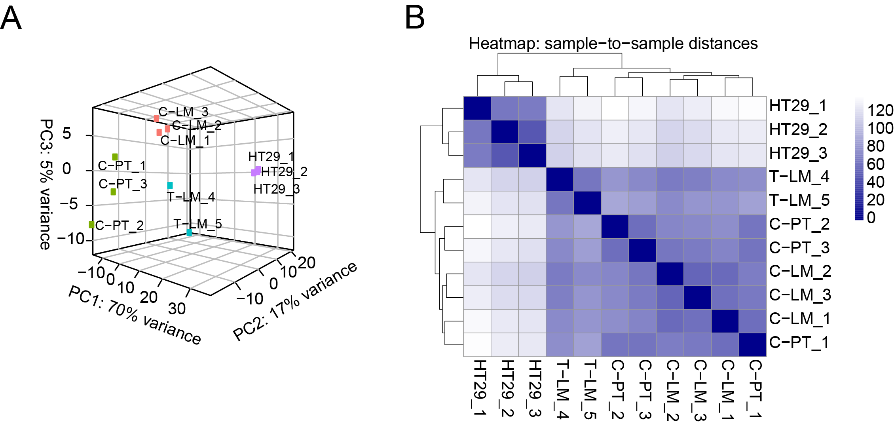


**Figure S2.** Quality control for rRNA-depleted library. (**A**) Principal component analysis of the samples from rRNA-depleted library. (**B**) Heatmap showing sample-to-sample distances from rRNA-depleted library.


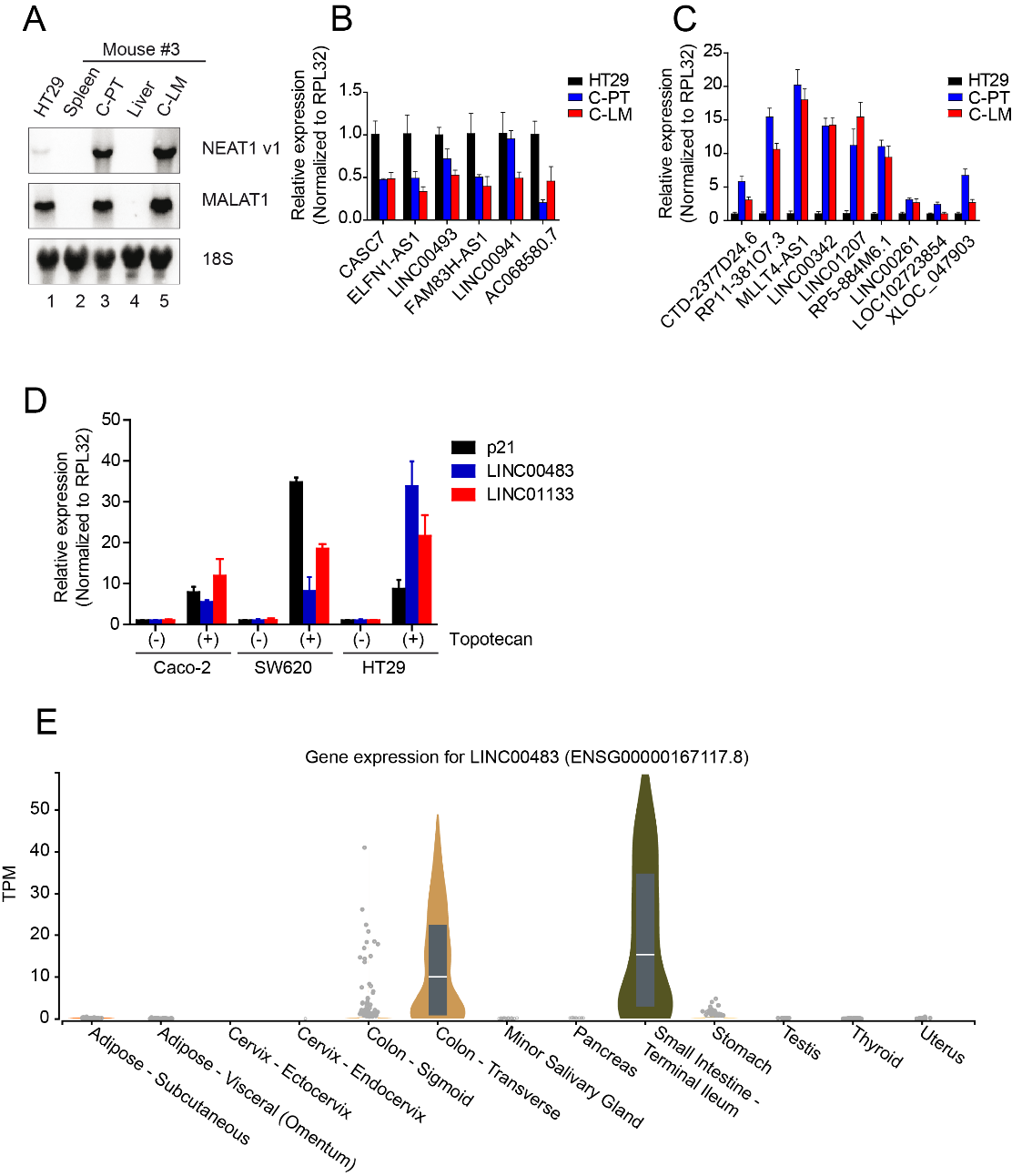


**Figure S3.** Validation of differentially expressed lincRNAs. (**A**) Northern blot validation of NEAT1 short isoform (NEAT1 v1) and MALAT1 in the primary tumor control group (C-PT), liver metastasis from control group (C-LM). 18S served as loading control. (**B**,**C**) Validation of downregulated and upregulated lncRNA from primary tumor control group (C-PT), liver metastasis from control group (C-LM) compared to HT29 cells by qPCR. (**D**) Relative expression of LINC00483 and LINC01133 in Caco-2, SW620 and HT29 cells 2 days after treatment with topotecan by qPCR. P21 served as positive control. (**E**) Gene expression for LINC00483 of different human tissue using GTExPortal web database.


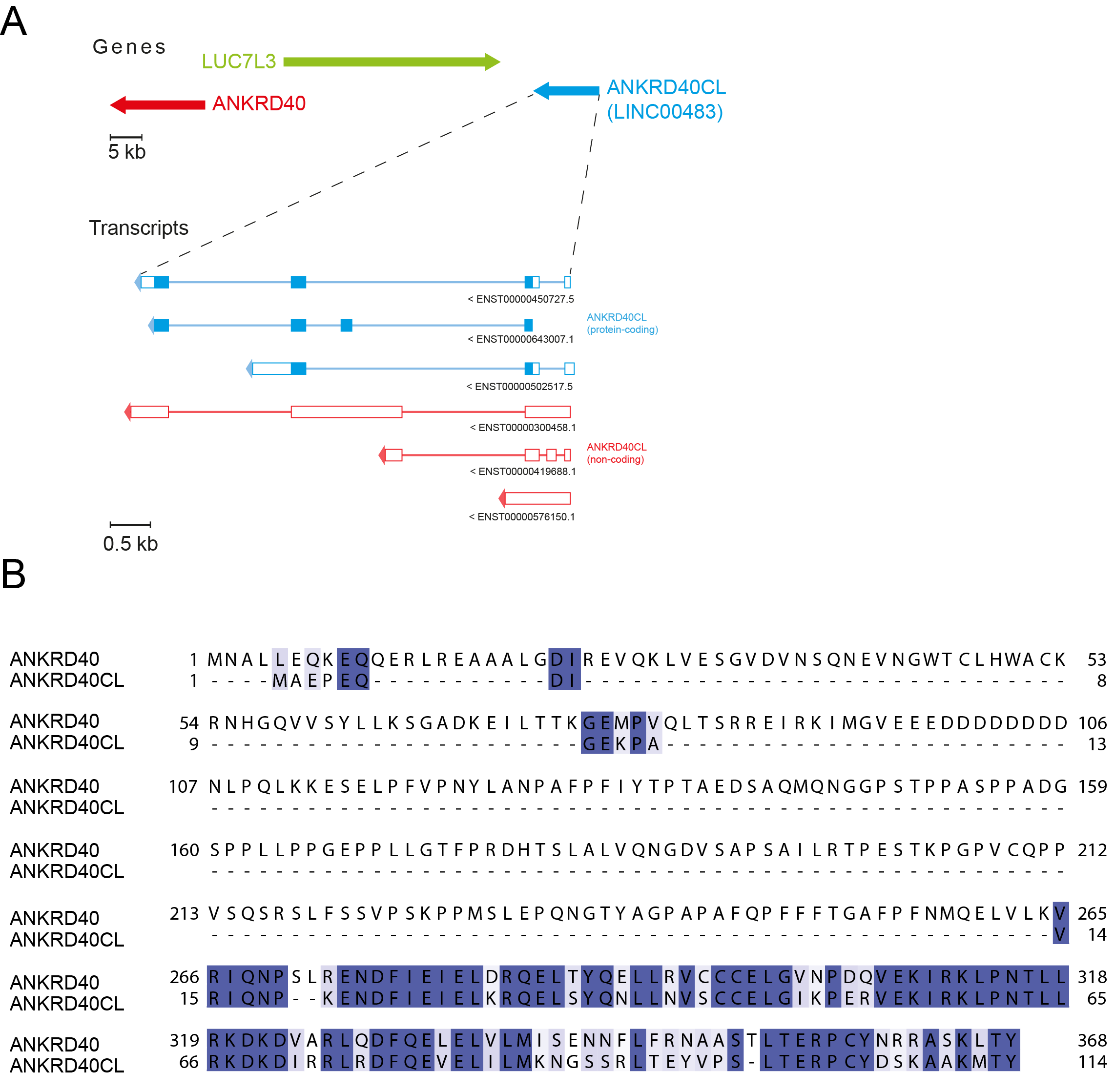


**Figure S4.** LINC00483 potentially encodes for a microprotein named ANKRD40CL. **(A**) Locus of LINC00483. LINC00483 encodes for several transcripts, some of which might encode for protein called ANKRD40CL. Sequence alignment of ANKRD40CL and ANKRD40. (B) Protein alignment of ANKRD40CL with ANDKRD40.


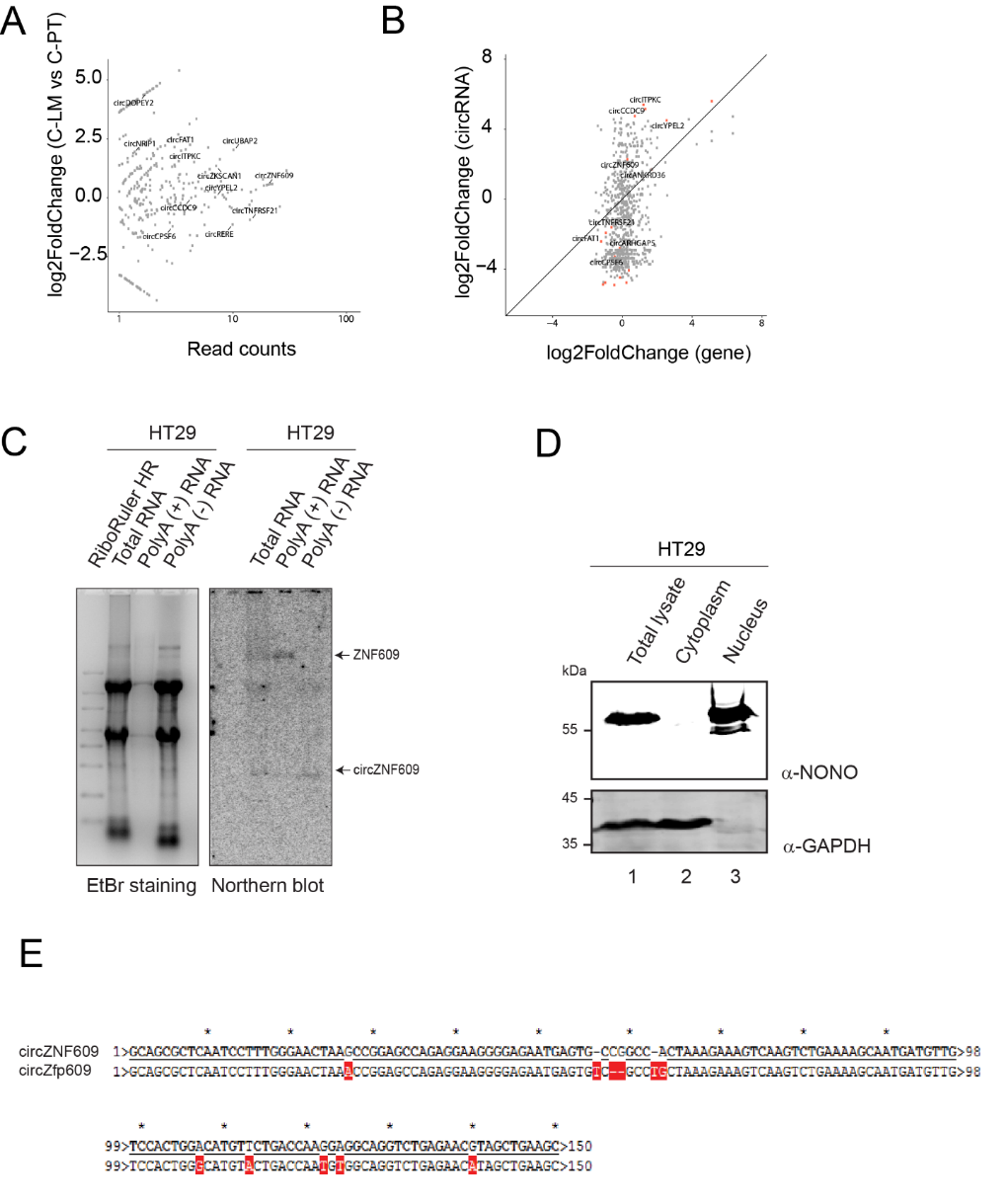


**Figure S5.** Further characterization of circZNF609. (**A**) Scatter plot of circRNA expression in C-LM versus C-PT. (**B**) Scatter plot of circRNA expression and linear transcript expression in liver metastasis versus HT29.hCG.Luc. Expression of some circRNAs is independent from its hots gene. (**C**) Northern blot of the fractionated polyA (+/−) RNA from HT29 cells. 20 µg of Total RNA or polyA (−) RNA and 2.5 µg of polyA (+) RNA was loaded on 1% MOPS Agarose gel. Northern blot using probes detected both ZNF609 and circZNF609 was performed. Northern blot analysis showed that circZNF609 is not enriched in the polyA (+) fraction as shown by ZNF609. (**D**) Western blot of the total lysate, nuclear and cytoplasmic fraction from HT29 cells. NONO and GAPDH served as nuclear and cytoplasmic marker, respectively. (**E**) Alignment of the amplicon’ sequence of circZNF609 and circZfp609 that amplified by qPCR primer.


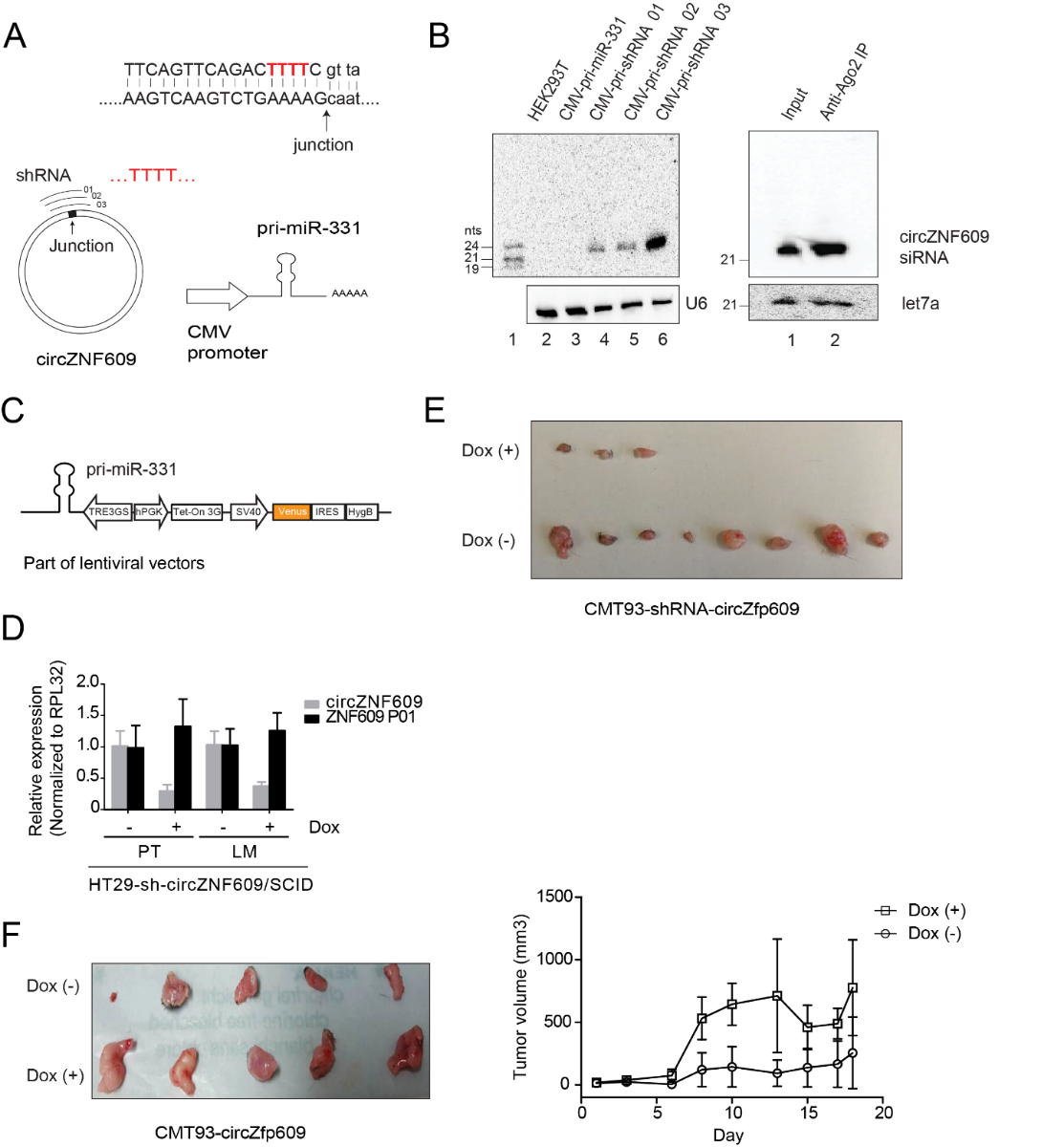


**Figure S6.** Methods for knockdown of circZNF609 and characterization the oncogenic role of circZNF609. (**A**) siRNA strategy for targeting circZNF609. Alignment of the siRNA with junction of circZNF609 is indicated. All three designed sequences spanning the junction of circZNF609 contain four Ts in a row in front of the stop signal, therefore lead to premature abortion of Pol-III transcription. Alternatively, artificial microRNA (amiRNA) construct based on pri-miR-331 driven by Pol-II promoter was developed. (**B**) HEK293T was transfected with the constructs consecutively expressing pri-miR-331 or different amiRNAs. 2 days post-transfection, RNA was harvested for northern blot analysis using probes targeting the desired sequence of siRNA targeting circZNF609 (siRNA 03). (**C**) Part of the lentiviral vectors used for generating HT29-sh-circZNF609 and HCT116-sh-circZNF609 cell lines (**D**) Relative expression of circZNF609 and ZNF609 from primary tumors (PT) and liver metastasis (LM) isolated from tumors developed in SCID mice injected with HT29-sh-circZNF609 cell lines. Data represents relative expression normalized to RPL32 from untreated animal (Dox (−), *n* = 4) and Doxycyline treated animal (Dox(+), *n* = 4). (**E**) In vivo data of tumor resected from the mice subcutaneously injected with CMT93-sh-circZfp609 upon doxycycline induction. The tumor development was calculated by measuring the tumor weight. (**F**) In vivo data from the mice overexpressing circZNF609 upon doxycycline induction (*n* = 10). Induction of circZNF609 by doxycycline increases tumor volume. Dox: Doxycyline.


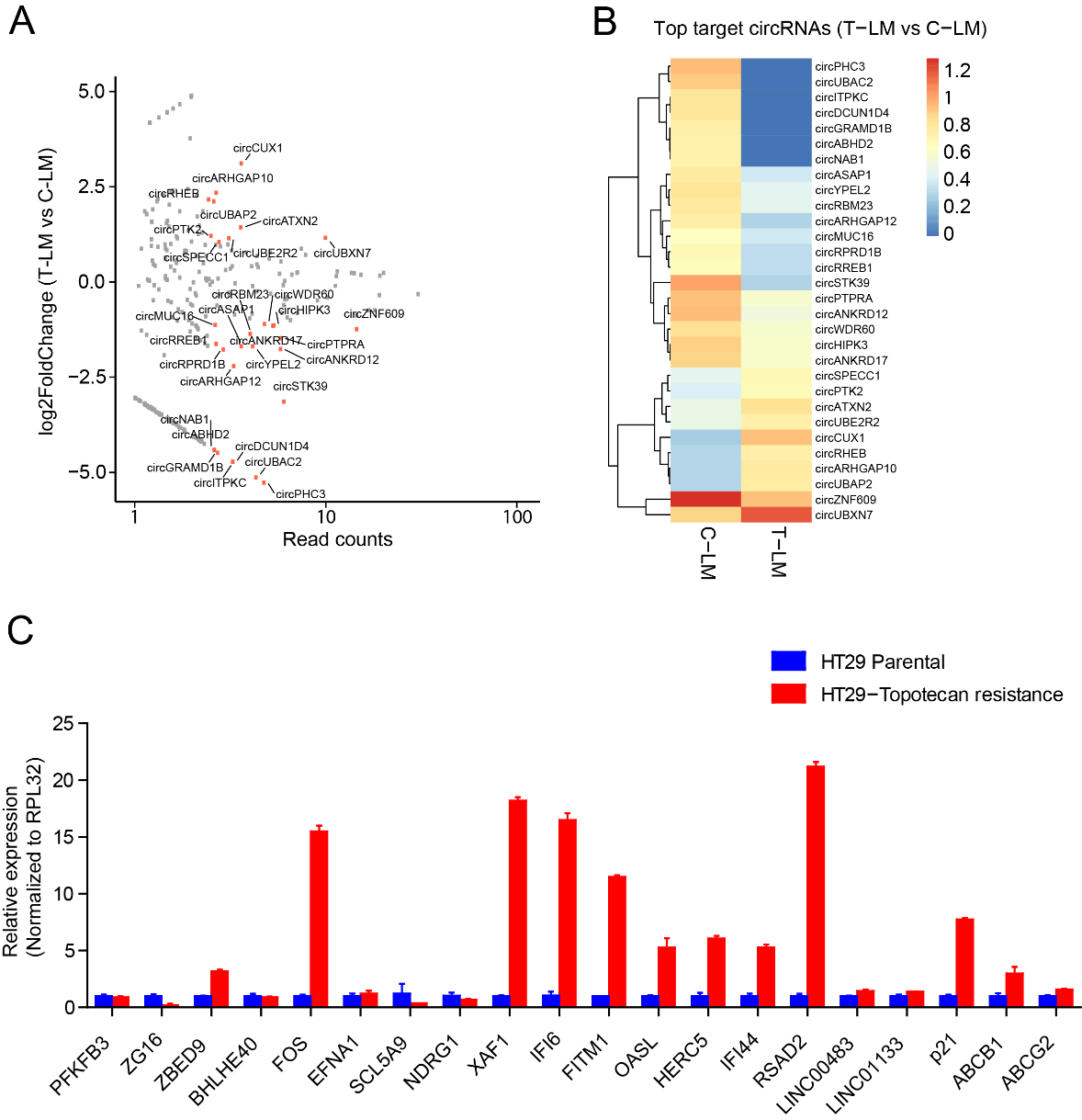


**Figure S7.** Differentially expressed circRNAs during LDM treatment and validation of RNA candidates in HT29-topotecan resistance cell line. (**A**) Scatter plot of circRNA expression in T-LM versus C-LM. (**B**) Heatmap of differentially expressed circRNAs in T-LM versus C-LM. The total number of reads supporting a particular head-to-tail junction was used as an absolute measure of circRNA abundance. (**C**) Examination of the validated candidates from HT29-topotecan resistance compared to HT29 parental cell line by qPCR.


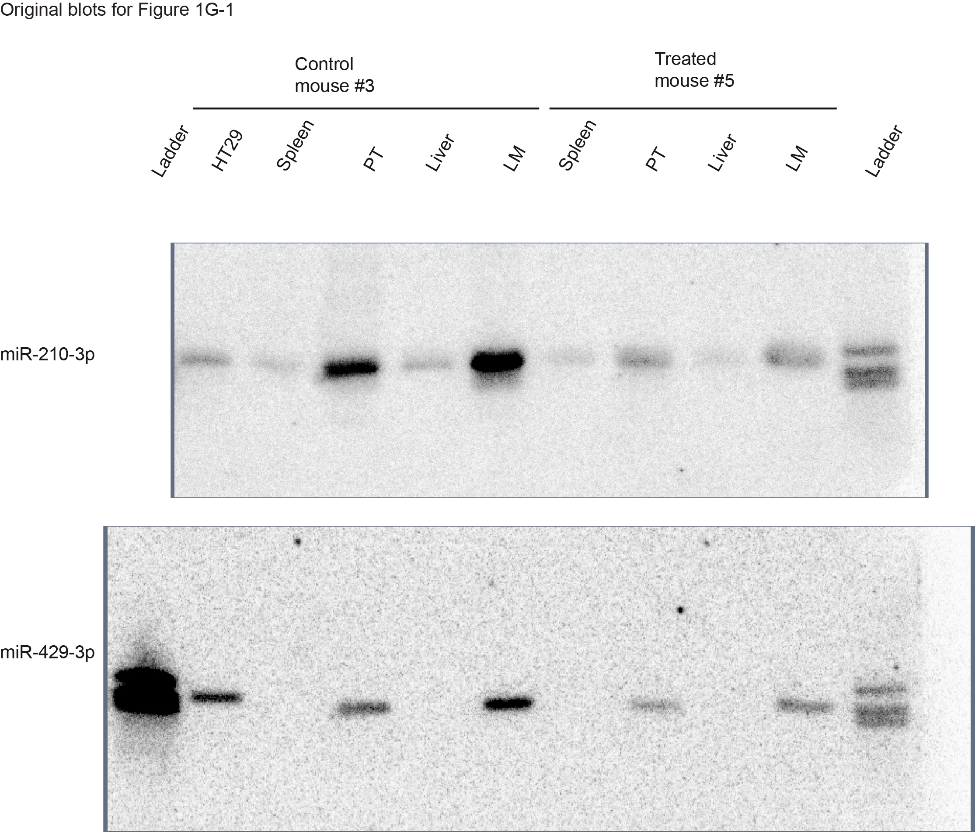


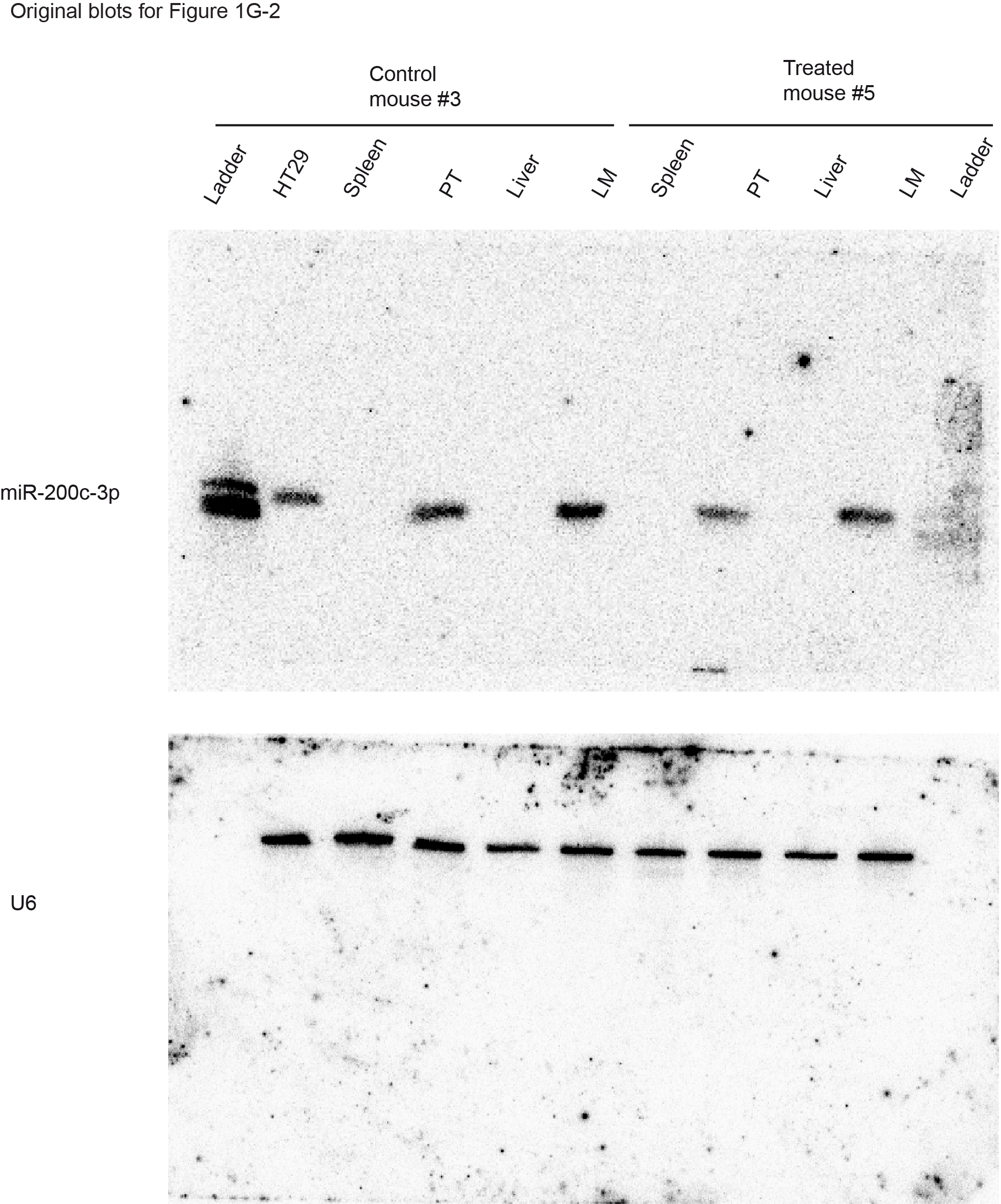


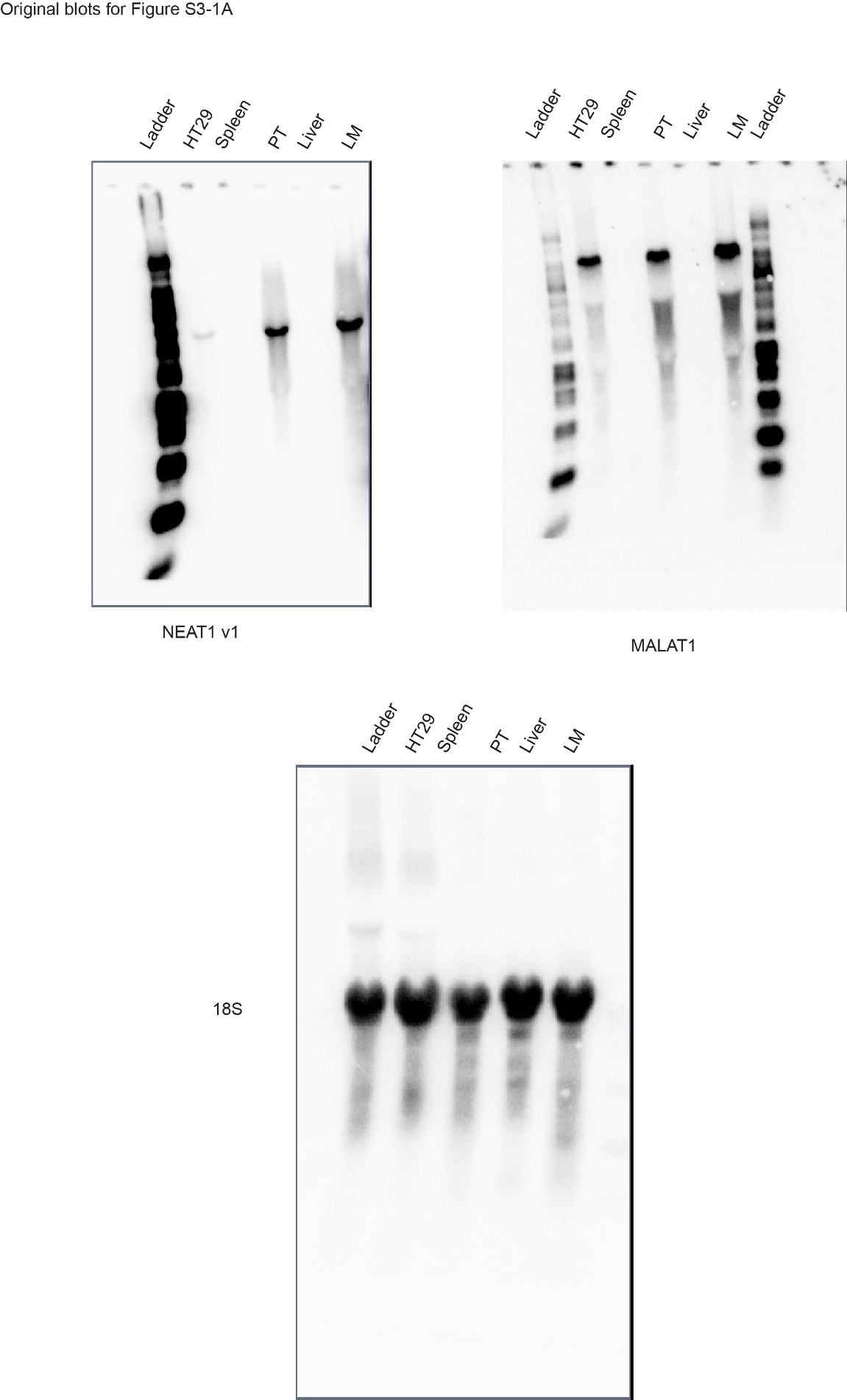


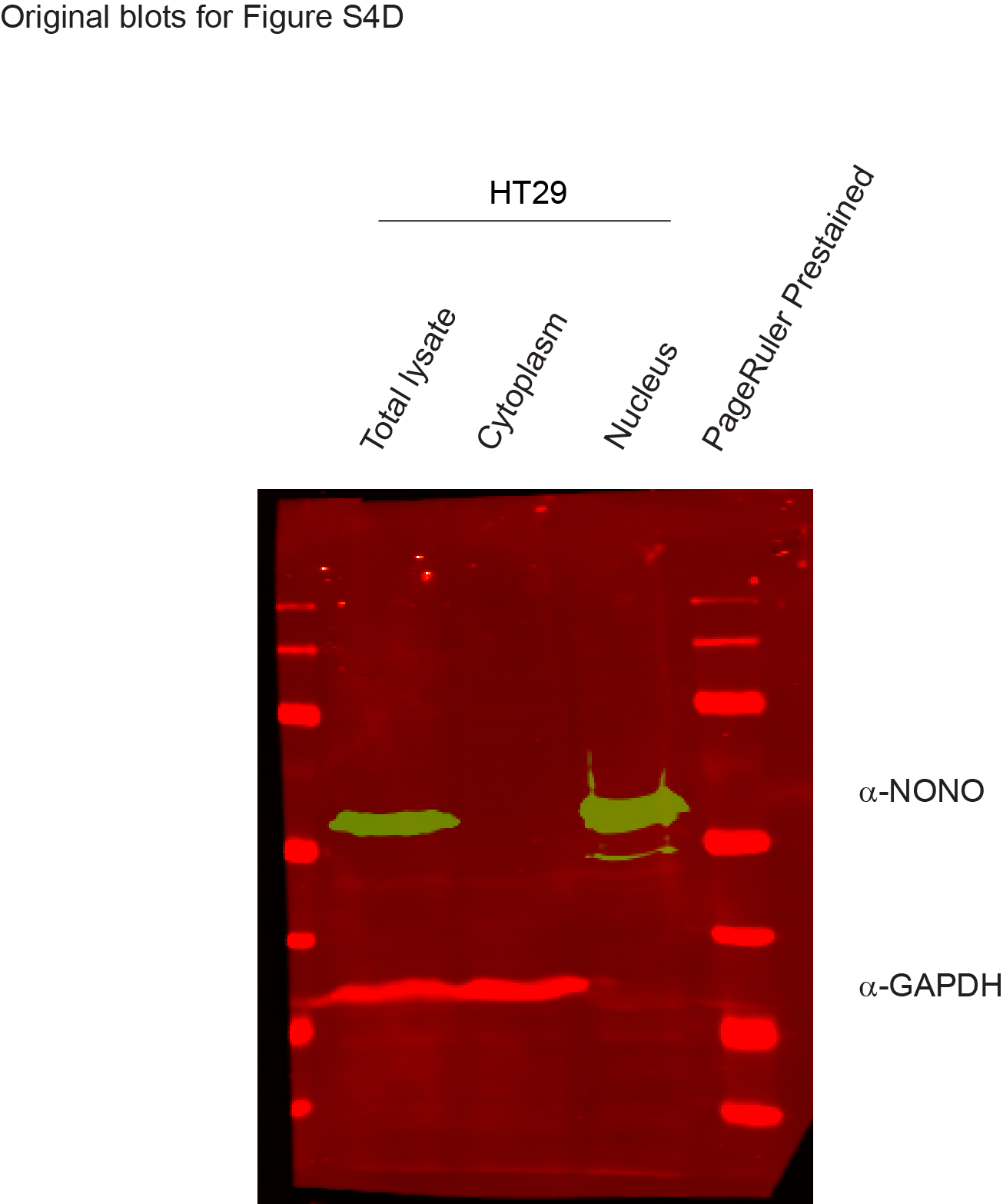


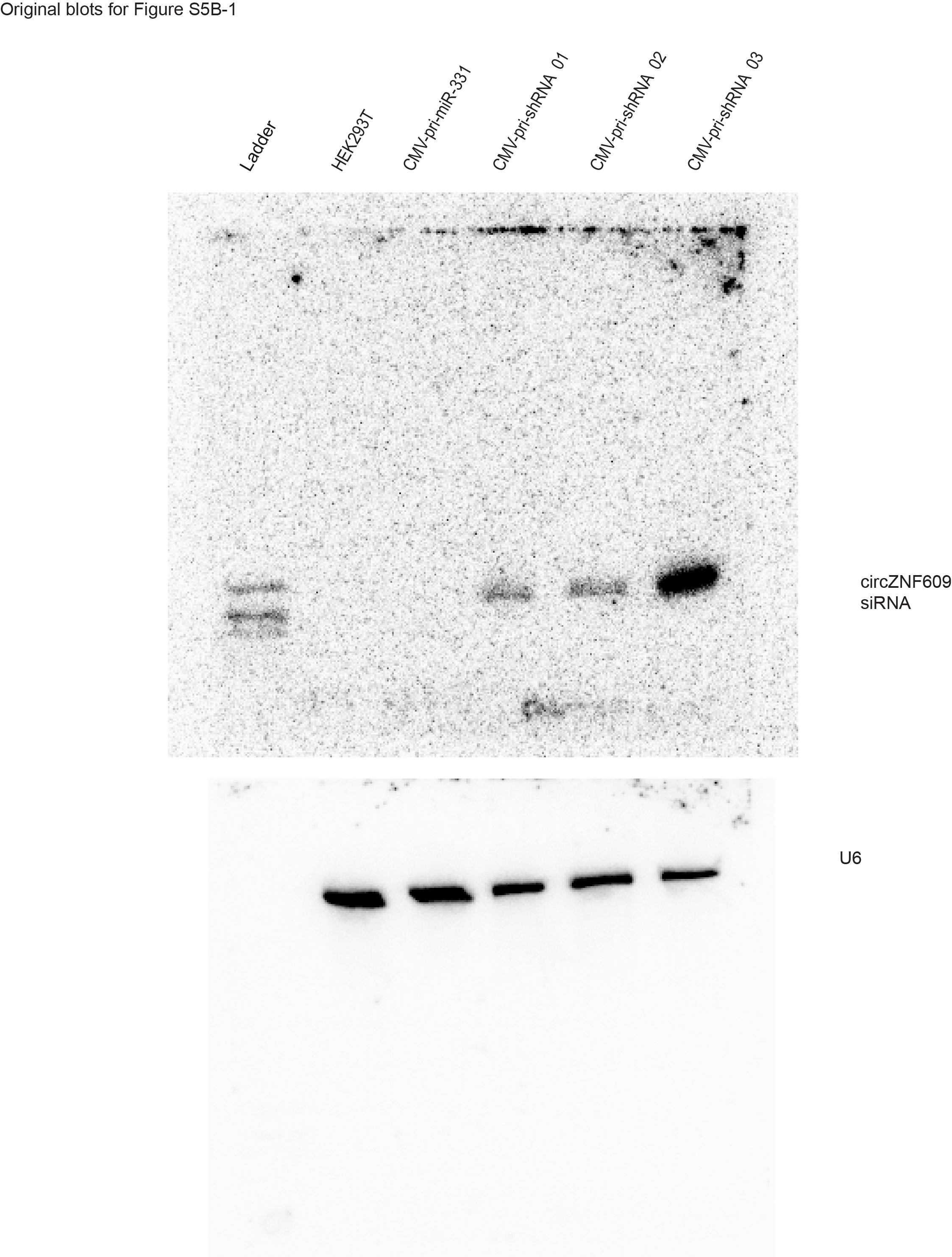


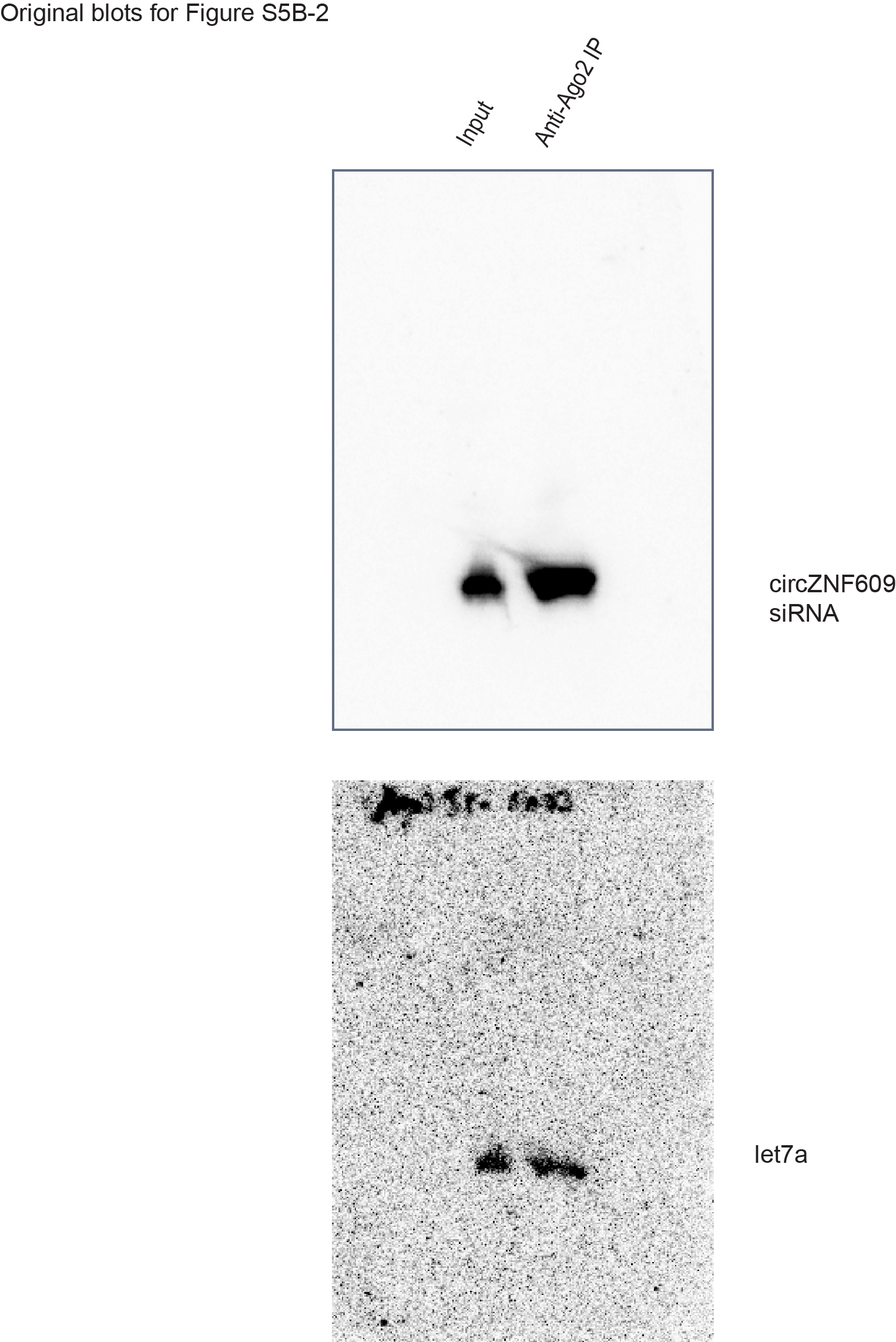


**Figure S8.** Uncropped Western Blots and Northern Blots.
